# Supplementary material for: FNIP1 abrogation promotes functional revascularization of ischemic skeletal muscle by driving macrophage recruitment
Source: Nat Commun. 2023 Nov 6;14:7136. doi: 10.1038/s41467-023-42690-9 (PMC10628247; doi:10.1038/s41467-023-42690-9)

Supplementary Information

FNIP1 abrogation promotes functional revascularization of ischemic skeletal muscle by driving macrophage recruitment

Zongchao Sun[1,#], Likun Yang[1,#], Abdukahar Kiram[1,#], Jing Yang[1,#], Zhuangzhuang Yang[2], Liwei Xiao[1], Yujing Yin[1], Jing Liu[1], Yan Mao[1], Danxia Zhou[1], Hao Yu[1], Zheng Zhou[1], Dengqiu Xu[1], Yuhuan Jia[1], Chenyun Ding[1], Qiqi Guo[1], Hongwei Wang[3], Yan Li[4], Li Wang[4], Tingting Fu[1,*], Shijun Hu[2,*] and Zhenji Gan[1,5,6,*]

[1]State Key Laboratory of Pharmaceutical Biotechnology and MOE Key Laboratory of Model Animal for Disease Study, Model Animal Research Center, Division of Spine Surgery, Department of Orthopedic Surgery, Nanjing Drum Tower Hospital, Medical School of Nanjing University, Nanjing University, Nanjing, China.
[2]Department of Cardiovascular Surgery of the First Affiliated Hospital & Institute for Cardiovascular Science, Collaborative Innovation Center of Hematology, State Key Laboratory of Radiation Medicine and Protection, Suzhou Medical College, Soochow University, Suzhou, China;
[3]Center for Translational Medicine and Jiangsu Key Laboratory of Molecular Medicine, Medical School of Nanjing University, Nanjing, China
[4]State Key Laboratory of Food Science and Technology, School of Food Science and Technology, Jiangnan University, Wuxi, China
[5]Jiangsu Key Laboratory of Molecular Medicine, Medical School of Nanjing University, Nanjing University, Nanjing, China.
[6]Chemistry and Biomedicine Innovation Center (ChemBIC), Nanjing University, Nanjing, China.

[#]These authors contributed equally: Zongchao Sun, Likun Yang, Abdukahar Kiram, Jing Yang
*To whom to address correspondence:
futt@nicemice.cn; shijunhu@suda.edu.cn; ganzj@nju.edu.cn

Contents:

1. Supplementary Figures 1-8.
2. Supplementary Tables 1.
3. Uncropped scans for Supplementary Figures 1-8.

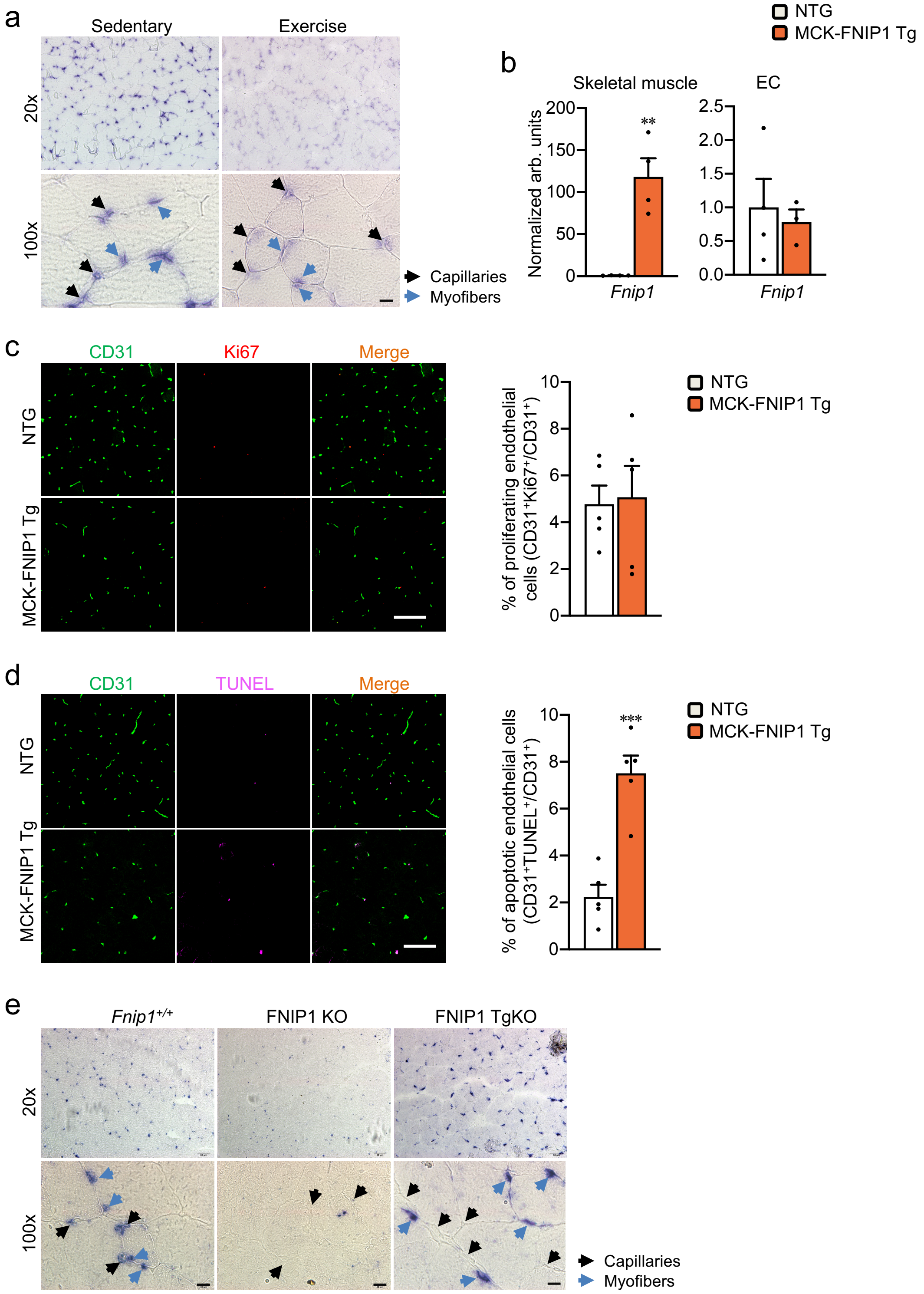

Supplementary Figure 1. Regulation of muscle angiogenesis by FNIP1. (**a**) Cross-sections from gastrocnemius (GC) muscle from 8-week-old WT C57BL/6J sedentary or exercise mice were used for RNA in situ hybridization using a probe unique for *Fnip1* (blue labeling). Representative images are shown. Blue arrows: Myofibers, Black arrows: Capillaries. Scale bar, 50 µm. Sedentary, n = 4; Exercise, n = 3 biologically independent mice. (**b**) Expression of *Fnip1* (qRT-PCR) in GC muscles (n

= 4 biologically independent mice, *P* value: 0.0018) and ECs (n = 3 biologically independent mice) isolated from muscles of 8-week-old NTG and FNIP1 Tg mice. (**c**) Representative images of immunostainings of CD31 (green) and Ki67 (red) co-staining in GC muscles from 8-week-old NTG and FNIP1 Tg mice. Scale bar, 100 µm. Quantification of proliferating endothelial cells. n = 5 biologically independent mice per group. (**d**) Representative images of CD31 (green) and TUNEL (magenta) co-staining in GC muscles from 8-week-old NTG and FNIP1 Tg mice. Scale bar, 100 µm. Quantification of apoptotic endothelial cells. n = 5 biologically independent mice per group. *P* value: 0.0004. (**e**) RNA in situ hybridization of *Fnip1* expression in 8-week-old $Fnip1^{+/+}$, FNIP1 KO and FNIP1 TgKO GC muscles cross-sections. Representative images are shown. Blue arrows: Myofibers, Black arrows: Capillaries. Scale bar, 50 µm. $Fnip1^{+/+}$, n = 4; FNIP1 KO, n = 3; FNIP1 TgKO, n = 3 biologically independent mice. All data are shown as the mean ± SEM. $^{**}P < 0.01$, $^{***}P < 0.001$ vs. NTG, determined by two-tailed unpaired Student's *t*-test. Source data are provided as a Source Data file.

Supplementary Figure 2

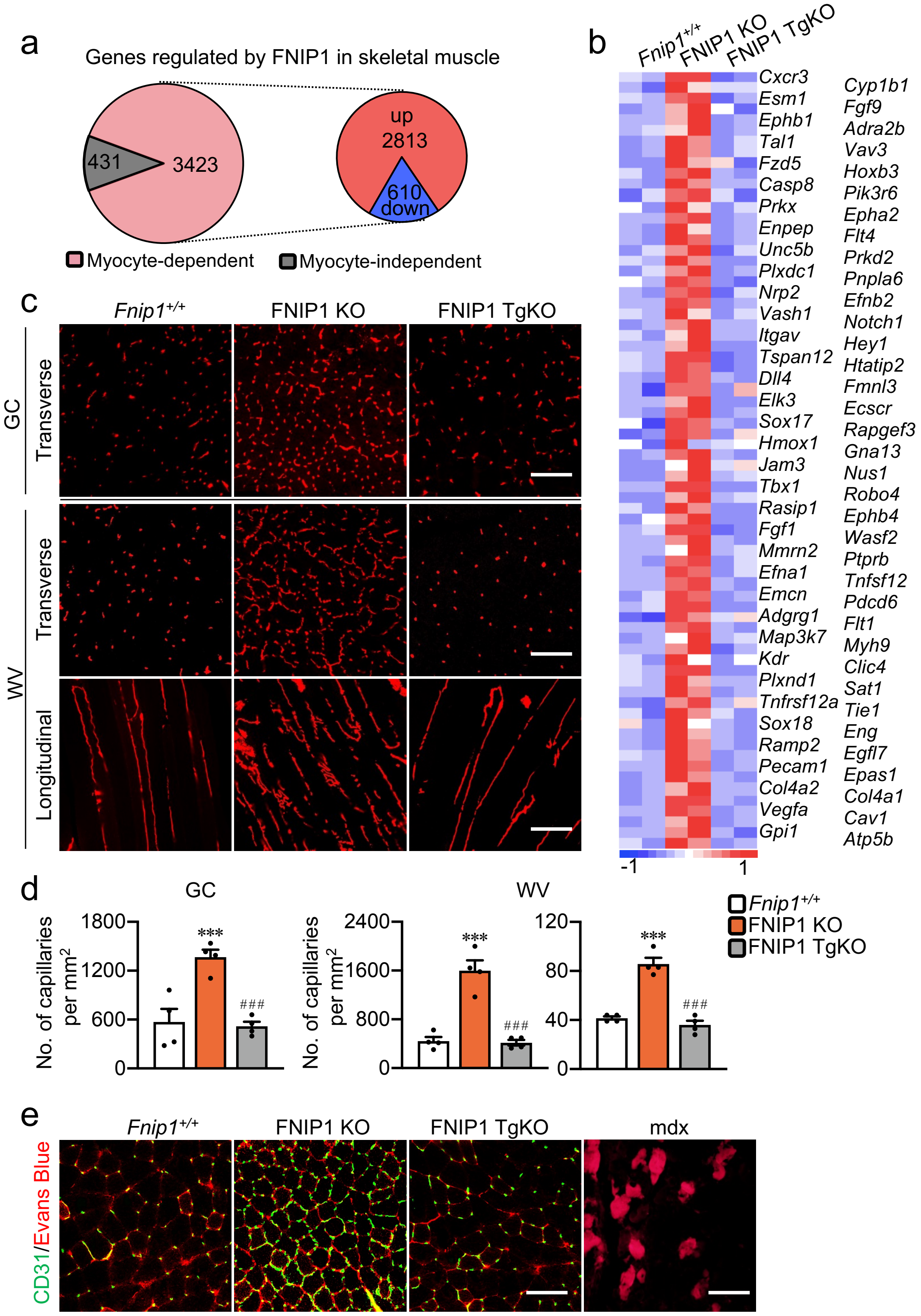

Supplementary Figure 2. FNIP1-dependent regulation of skeletal muscle angiogenesis. (**a**) Schematic of identification of genes regulated by FNIP1 with the cutoff criteria of a fold change greater than 1.5 (either direction) and a significant $P < 0.05$. (**b**) Heatmap analysis of FNIP1-regulated genes in skeletal muscle. n = 2

independent samples per group. Color scheme for fold change is provided. (**c**) Representative confocal images of microsphere perfused 8-week-old indicated mice GC and WV muscles. Scale bar, 100 µm. (**d**) Quantification of capillaries per mm^2 in (**c**). n = 4 biologically independent mice per group. **P* value: 0.0007, < 0.0001, < 0.0001. #*P* value: 0.0005, < 0.0001, < 0.0001. (**e**) Evans blue dye infiltration test to examine vascular leak in the GC muscles of 14-week-old indicated mice. n = 4 biologically independent mice per group. All data are shown as the mean ± SEM. ****P* < 0.001 vs. corresponding *Fnip1*$^{+/+}$ controls, ###*P* < 0.001 vs. FNIP1 KO, determined by one-way ANOVA coupled to Fisher's least significant difference (LSD) post-hoc test. Source data are provided as a Source Data file.

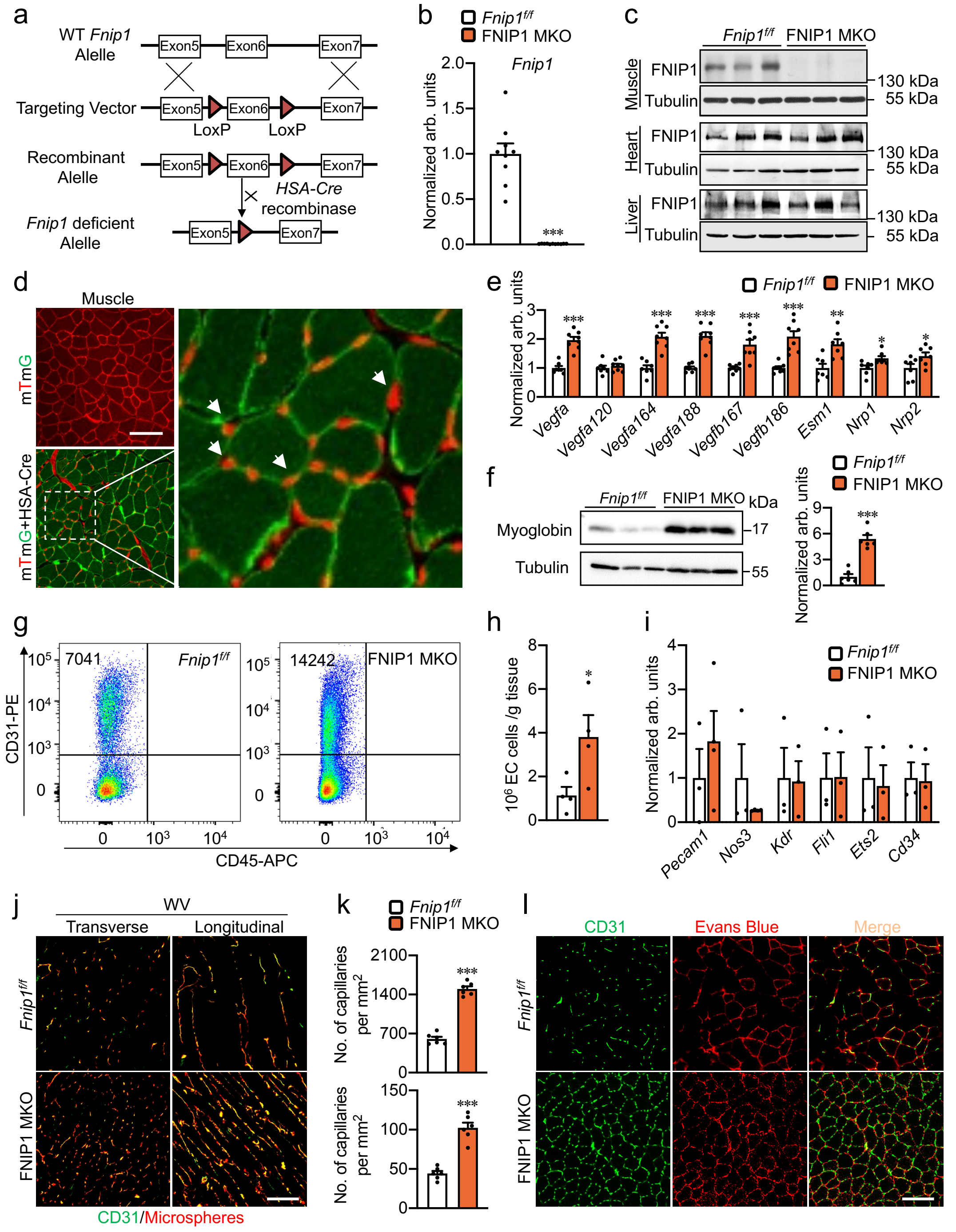

Supplementary Figure 3. Myofiber-specific ablation of FNIP1 promotes the formation of patent, functional blood vessels. (**a**) Schematic showing the generation of myofiber-specific FNIP1 KO mice. (**b**) qRT-PCR analysis of *Fnip1* mRNA levels in the TA muscle from 14-week-old *Fnip1$^{f/f}$* and FNIP1 MKO mice. *Fnip1$^{f/f}$*, n = 9; FNIP1 MKO, n =11 biologically independent mice. *P* value: < 0.0001. (**c**) Representative western blot analysis of FNIP1 protein expression in muscle, heart, and liver from 14-week-old indicated genotypes. n = 3 biologically independent mice. (**d**) Confocal images of muscle from Rosa26mTmG cell fate-tracing mouse demonstrated that HSA-Cre activity is restricted to myofibers and is largely absent in

non-muscle cells. n = 4 biologically independent mice. (**e**) Expression of genes (qRT-PCR) associated with angiogenesis in GC muscles from the indicated genotypes. n = 8 biologically independent mice per group. *P* value: < 0.0001, < 0.0001, < 0.0001, 0.0003, < 0.0001, 0.0034, 0.0154, 0.0455. (**f**) Representative Western blot analysis of Myoglobin protein expression in GC muscles from indicated genotypes. Quantification of Myoglobin/Tubulin. n = 6 biologically independent mice. *P* value: 0.0001. (**g**) Representative flow cytometric analysis of $CD31^+$ $CD45^-$ cells in muscles of 10-week-old *Fnip1*$^{f/f}$ and FNIP1 MKO mice. (**h**) Total number of $CD31^+CD45^-$ ECs in muscle determined by flow cytometry. n = 4 biologically independent mice. *P* value: 0.0476. (**i**) Expression of endothelial cell specific marker genes (qRT-PCR) in isolated ECs from the indicated genotypes. n = 3 biologically independent mice. (**j**) Representative confocal images of microsphere perfused indicated mice WV muscles. Scale bar, 100 µm. (**k**) Quantification of capillaries per mm^2 in (**j**). n = 6 biologically independent mice. *P* value: < 0.0001. (**l**) Evans blue dye infiltration test to examine vascular leak in the GC muscles of 8-week-old indicated mice. n = 5 biologically independent mice. All data are shown as the mean ± SEM. *$P < 0.05$, **$P < 0.01$, ***$P < 0.001$ vs. corresponding *Fnip1*$^{f/f}$ controls determined by two-tailed unpaired Student's *t*-test. Source data are provided as a Source Data file.

Supplementary Figure 4

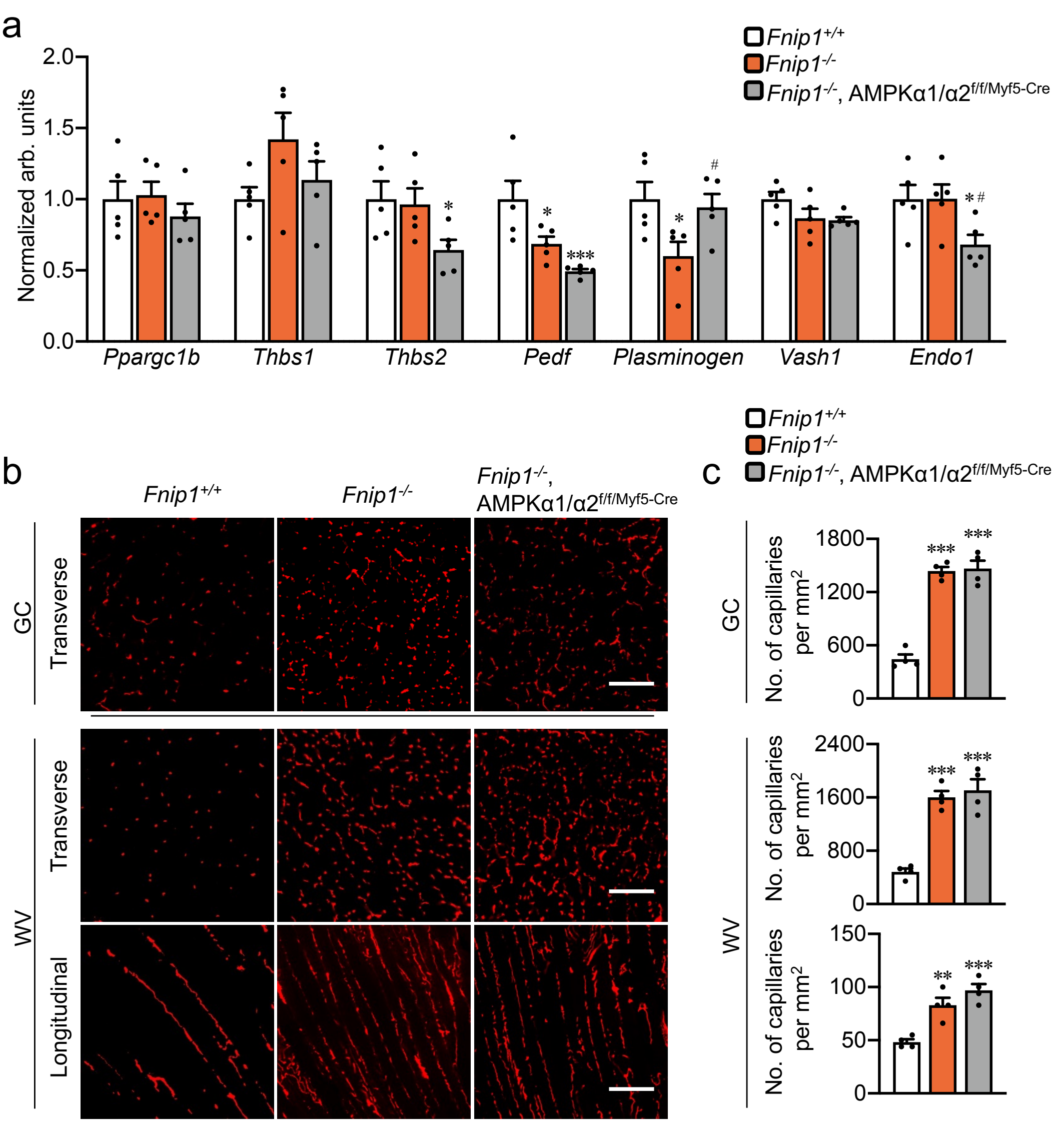

Supplementary Figure 4. AMPK-independent regulation of muscle angiogenesis by FNIP1. (**a**) Expression of antiangiogenic genes (qRT-PCR) in GC muscles from the 8-week-old indicated genotypes. n = 5 biologically independent mice per group. **P* value: 0.0346, 0.0176, 0.0008, 0.0197, 0.0295, #*P* value: 0.0396, 0.0283. (**b**) Representative confocal images of microsphere perfused indicated mice GC and WV muscles. Scale bar, 100 µm. (**c**) Quantification of capillaries per mm^2 in (**b**). n = 4 biologically independent mice per group. *P* value: < 0.0001, < 0.0001, < 0.0001, < 0.0001, 0.0015, 0.0001. All data are shown as the mean ± SEM. **P* < 0.05, ***P* < 0.01, ****P* < 0.001 vs. corresponding *Fnip1*$^{+/+}$ controls, #*P* < 0.05 vs. *Fnip1*$^{-/-}$, determined by one-way ANOVA coupled to Fisher's least significant difference (LSD) post-hoc test. Source data are provided as a Source Data file.

Supplementary Figure 5

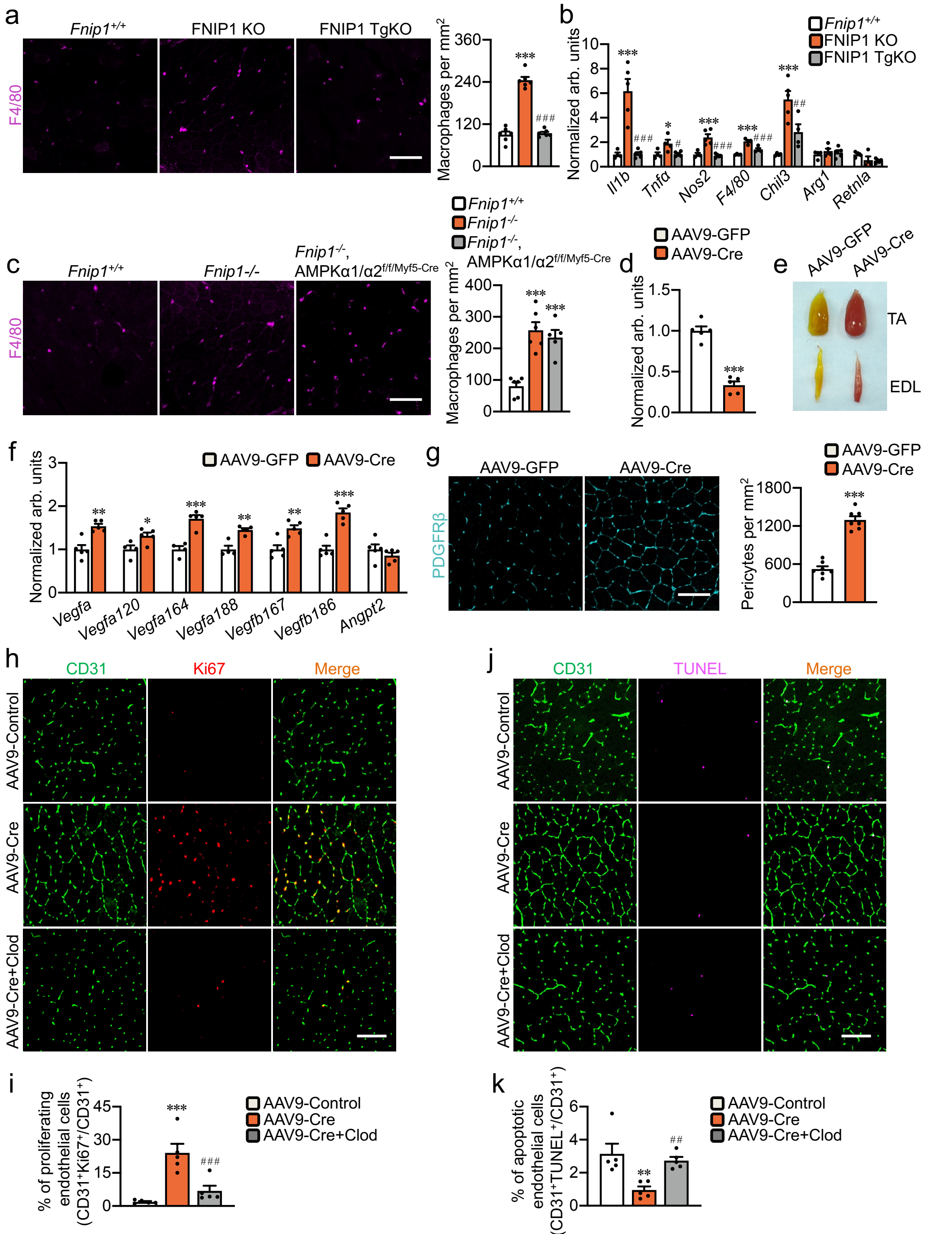

Supplementary Figure 5. FNIP1 regulates muscle angiogenesis through macrophage recruitment. (**a**, **c**) Representative images of F4/80 immunofluorescent staining in GC muscles from 8-week-old indicated mice. Scale bar, 100 μm. Quantification of F4/80-positive macrophages per mm^2. (**a**) n = 6 biologically independent mice, **P* value: < 0.0001. #*P* value: < 0.0001. (**c**) $Fnip1^{+/+}$, $Fnip1^{-/-}$, n = 6, $Fnip1^{-/-}$, AMPKα1/α2$^{f/f/Myf5\text{-}Cre}$, n = 5 biologically independent mice. *P* value: < 0.0001, 0.0002. (**b**) Expression of macrophage activation genes (qRT-PCR) associated with angiogenesis in GC muscles from the 8-week-old indicated genotypes. n = 5

biologically independent mice. **P* value: 0.0001, 0.0127, 0.0003, < 0.0001, 0.0002. #*P* value: < 0.0001, 0.0117, < 0.0001, 0.0007, 0.0078. (**d**) qRT-PCR analysis of *Fnip1* mRNA levels in TA muscles from $Fnip1^{f/f}$ mice injected with AAV9-Cre or control viruses. n = 5 biologically independent mice. *P* value: < 0.0001. (**e**) Representative TA and EDL muscles from indicated mice. (**f**) Expression of genes (qRT-PCR) associated with angiogenesis in TA muscles from the indicated mice. n = 5 biologically independent mice. *P* value: 0.0016, 0.0309, 0.0005, 0.0013, 0.0038, 0.00015. (**g**) Representative images of PDGFRβ immunofluorescent staining in TA muscles from indicated mice. Scale bar, 100 μm. Quantification of pericytes per mm^2. n = 7 biologically independent mice. *P* value: < 0.0001. (**h**) Representative images of CD31 (green) and Ki67 (red) co-staining in TA muscles from indicated mice. Scale bar, 100 μm. (**i**) quantification of proliferating endothelial cells. n = 5 mice per group. **P* value: 0.0001. #*P* value: 0.0009. (**j**) Representative images of CD31 (green) and TUNEL (magenta) co-staining in TA muscles from indicated mice. Scale bar, 100 μm. (**k**) quantification of apoptotic endothelial cells. n = 5 mice per group. **P* value: 0.0023. #*P* value: 0.0085. All data are shown as the mean ± SEM. **P* < 0.05, ***P* < 0.01, ****P* < 0.001 vs. corresponding $Fnip1^{+/+}$ or AAV9-GFP controls, #*P* < 0.05, ##*P* < 0.01, ###*P* < 0.001 vs. FNIP1 KO, determined by two-tailed unpaired Student's *t*-test (**d**, **f** and **g**) or one-way ANOVA (**a-c**, **i** and **k**) coupled to Fisher's least significant difference (LSD) post-hoc test. Source data are provided as a Source Data file.

Supplementary Figure 6

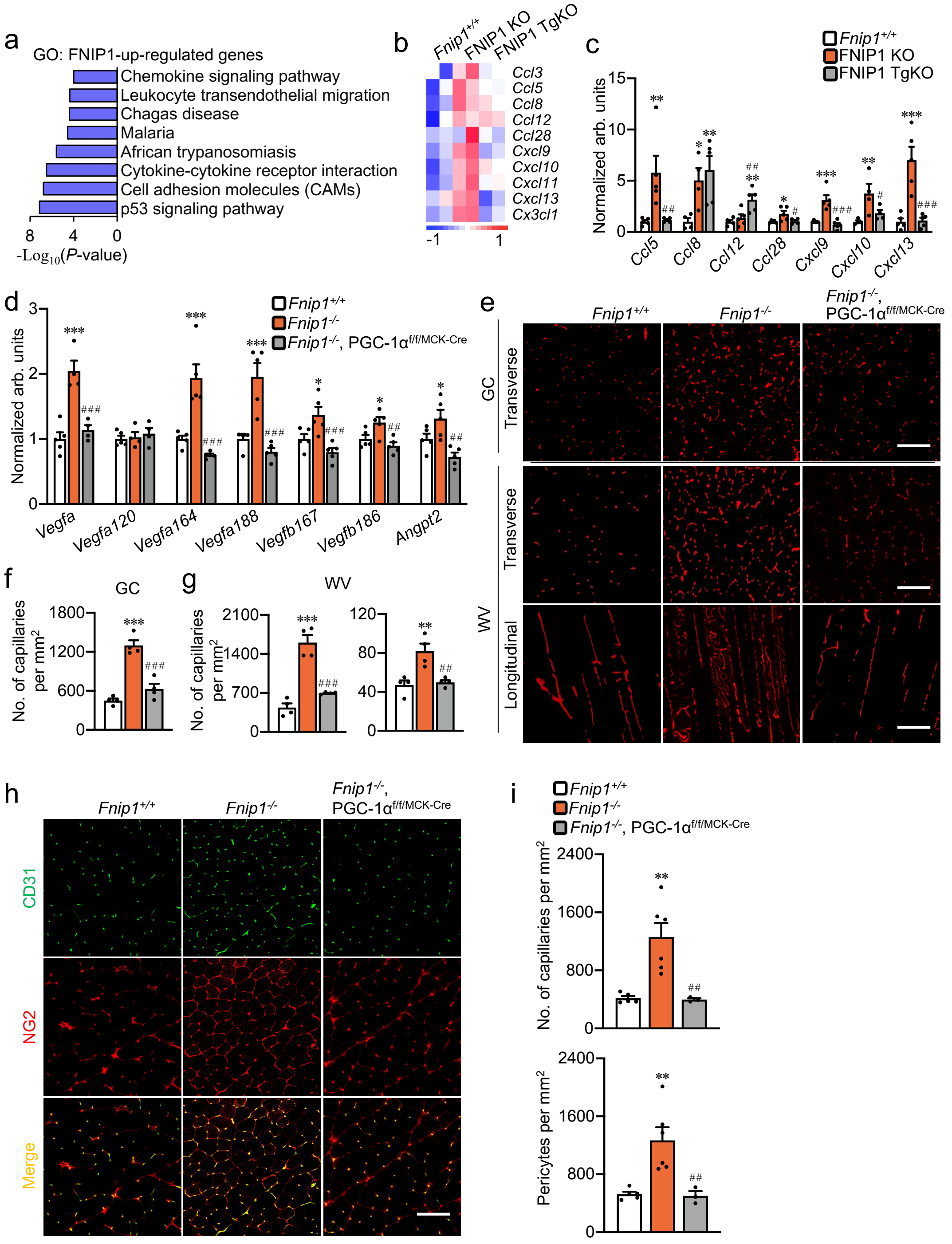

Supplementary Figure 6. Muscle FNIP1 deficiency induces PGC-1α to activate chemokine gene expression. (**a**) GO enrichment analysis (KEGG pathway) of gene transcripts regulated only in FNIP1 KO but not in FNIP1 TgKO muscles, with the top 8 terms shown. (**b**) Heatmap analysis of chemokine genes regulated by FNIP1. n = 2 independent samples per group. Color scheme for fold change is provided. (**c**) Expression of genes (qRT-PCR) involved in Chemokine signaling pathway in GC muscles from the 8-week-old indicated genotypes. n = 5 biologically independent mice. *P value: 0.0042, 0.0368, 0.0098, 0.0032, 0.0169, 0.0003, 0.0097, 0.0002. #P

value: 0.0052, 0.0067, 0.028, < 0.0001, 0.0474, 0.0002. (**d**) Expression of genes (qRT-PCR) associated with angiogenesis in GC muscles from the indicated genotypes. n = 5 biologically independent mice. *P value: < 0.0001, 0.0002, 0.0003, 0.0165, 0.0201, 0.0431. #P value: 0.0003, <0.0001, < 0.0001, 0.001, 0.0025, 0.0011.(**e**) Representative confocal images of microsphere perfused 8-week-old indicated mice GC and WV muscles. Scale bar, 100 µm. (**f**, **g**) Quantification of capillaries per mm^2 in (**e**). n = 4 biologically independent mice. *P value: < 0.0001, < 0.0001, 0.0015. #P value: < 0.0001, < 0.0001, 0.0025. (**h**) Representative images of CD31 (green) and NG2 (red) co-staining in muscles from indicated female mice. (**i**) Quantification of capillaries and pericytes in (**h**). *Fnip1*$^{+/+}$, n = 5; *Fnip1*$^{-/-}$, n = 6; *Fnip1*$^{-/-}$, PGC-1$\alpha^{f/f/MCK\text{-}Cre}$, n = 3 biologically independent mice. *P value: 0.0013, 0.0022. #P value: 0.0032, 0.0049. All data are shown as the mean ± SEM. *P < 0.05, **P < 0.01, ***P < 0.001 vs. corresponding controls, #P < 0.05, ##P < 0.01, ###P < 0.001 versus FNIP1 KO, determined by one-way ANOVA coupled to Fisher's least significant difference (LSD) post-hoc test. Source data are provided as a Source Data file.

Supplementary Figure 7

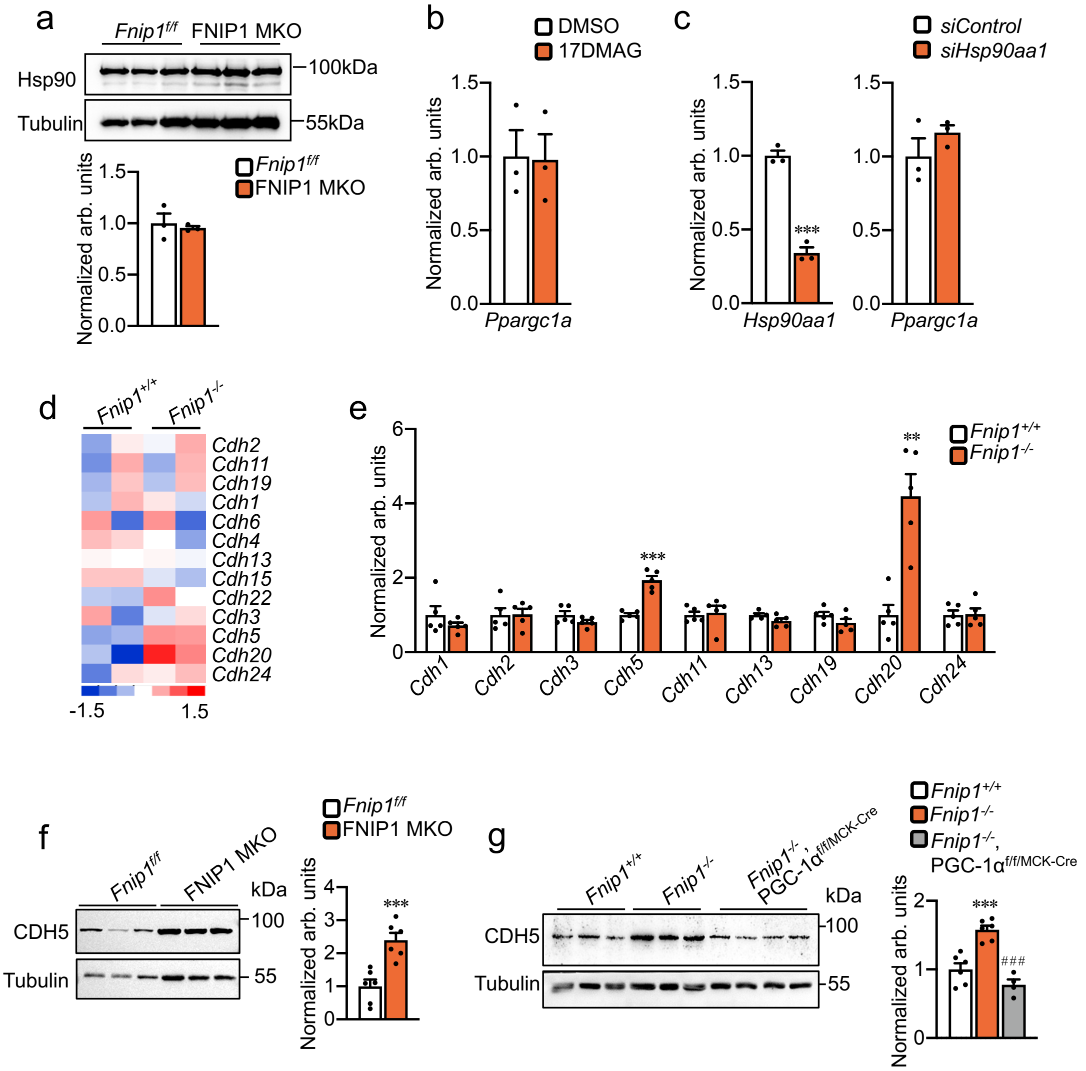

Supplementary Figure 7. Regulation of VE-cadherin gene CDH5 expression by FNIP1/PGC-1α. (**a**) Western blot analysis of Hsp90 protein levels in GC muscles from 14-week-old *Fnip1$^{f/f}$* and FNIP1 MKO mice. Quantification of the Hsp90/Tubulin. n = 3 biologically independent mice. (**b**) Expression of *Ppargc1a* (qRT-PCR) in C2C12 myotubes treated with DMSO or 17DMAG for 24 hours. n = 3 independent experiments. (**c**) Expression of *Hsp90aa1* and *Ppargc1a* (qRT-PCR) in C2C12 cells transfected with control siRNA for *Hsp90aa1* siRNA. n = 3 independent experiments. *P* value: 0.0002. (**d**) Heatmap analysis of VE-cadherin family gene expression in *Fnip1*$^{f/f}$ and FNIP1 MKO mice. (**e**) Expression of VE-cadherin family genes (qRT-PCR) in GC muscles from 14-week-old *Fnip1*$^{-/-}$ and *Fnip1*$^{+/+}$ mice. n = 5 biologically independent mice. *P* value: < 0.0001, 0.0013. (**f**, **g**) Western blot analysis of CHD5 protein levels in WV muscles from 8-week-old indicated mice. Quantification of the CDH5/Tubulin. *Fnip1$^{f/f}$*, n = 6; FNIP1 MKO, n = 6; *Fnip1*$^{+/+}$, *Fnip1*$^{-/-}$, n = 6; *Fnip1*$^{-/-}$, PGC-1α$^{f/f/MCK\text{-}Cre}$, n = 4 biologically independent mice. *P* value: 0.0009 (**f**). **P* value: < 0.0001, #*P* value: < 0.0001 (**g**). All data are shown as the mean ± SEM. ***P* < 0.01, ****P* < 0.001 vs. corresponding controls, ###*P* < 0.001

vs. *Fnip1*$^{-/-}$, determined by two-tailed unpaired Student's *t*-test (**a**, **b**, **c**, **e** and **f**) or one-way ANOVA (**g**) coupled to Fisher's least significant difference (LSD) post-hoc test. Source data are provided as a Source Data file.

Supplementary Figure 8

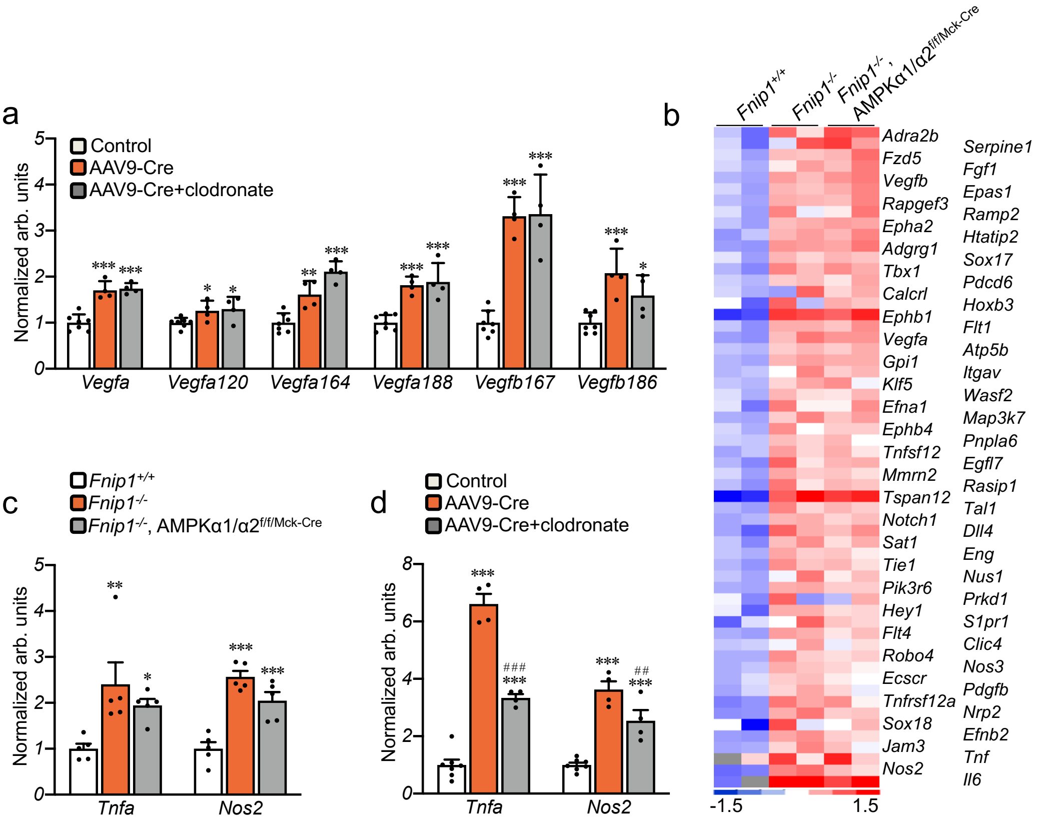

Supplementary Figure 8. Factors from outside the VEGF family could be involved in the activation of angiogenesis by FNIP1 deficiency. (**a**) Expression of genes (qRT-PCR) associated with angiogenesis in TA muscles from 8-week-old *Fnip1*$^{f/f}$ mice injected with AAV9-Cre or control viruses following clodronate treatment. Control, n = 7; AAV-Cre, n = 4; AAV-Cre+clodronate, n = 4 biologically independent mice per group. *P* value: < 0.0001, < 0.0001, 0.0496, 0.0269, 0.0014, < 0.0001, 0.0003, 0.0001, < 0.0001, < 0.0001, 0.0007, 0.0285. (**b**) Heatmap analysis of angiogenesis gene expression in skeletal muscle from indicated mice. (**c**) Expression of *Tnfa* and *Nos2* (qRT-PCR) in GC muscles from the indicated mice. n = 5 biologically independent mice per group. *P* value: 0.0057, 0.0438, <0.0001, 0.0005. (**d**) Expression of *Tnfa* and *Nos2* (qRT-PCR) in TA muscles from *Fnip1*$^{f/f}$ mice injected with AAV9-Cre or control viruses following clodronate treatment. Control, n = 7; AAV-Cre, n = 4; AAV-Cre+clodronate, n = 4 biologically independent mice per group. **P* value: <0.0001, <0.0001, <0.0001, 0.0003. #*P* value: < 0.0001, 0.0086. All data are shown as the mean ± SEM. **P* < 0.05, ***P* < 0.01, ****P* < 0.001 vs. corresponding controls, ##*P* < 0.01, ###*P* < 0.001 vs. AAV9-Cre, determined by one-way ANOVA coupled to Fisher's least significant difference (LSD) post-hoc test. Source data are provided as a Source Data file.

Supplementary Table 1. RT-qPCR primers

Table S1 RT-qPCR primers

Mouse Gene	*Forward*	*Reverse*
36b4	*5'-ATCCCTGACGCACCGCCGTGA*	*5'-TGCATCTGCTTGGAGCCCACGTT*
Gapdh	*5'-CATCACTGCCACCCAGAAGACTG*	*5'-ATGCCAGTGAGCTTCCCGTTCAG*
Angpt2	*5'-CTCACCACCAGTGGCATCTA*	*5'-CCCACGTCCATGTCACAGTA*
Arg1	*5'-CTCCAAGCCAAAGTCCTTAGAG*	*5'-GGAGCTGTCATTAGGGACATCA*
Cd34	*5'- AGGACAGCAGTAAGACCACACC*	*5'- GTGTGGAGTTCCAGAGCCTGAA*
Ccl4	*5'-ACCCTCCCACTTCCTGCTGTTT*	*5'-CTGTCTGCCTCTTTTGGTCAGG*
Ccl5	*5'-CCTGCTGCTTTGCCTACCTCTC*	*5'-ACACACTTGGCGGTTCCTTCGA*
Ccl8	*5'-GGGTGCTGAAAAGCTACGAGAG*	*5'-GGATCTCCATGTACTCACTGACC*
Ccl12	*5'-GCTACAGGAGAATCACAAGCAGC*	*5'-ACGTCTTATCCAAGTGGTTTATGG*
Ccl28	*5'-GTTCATGCAGCATCCAGAGAGC*	*5'-TCTGAGGCTCTCATCCACTGCT*
Cxcl9	*5'-CCTAGTGATAAGGAATGCACGATG*	*5'-CTAGGCAGGTTTGATCTCCGTTC*
Cxcl10	*5'-ATCATCCCTGCGAGCCTATCCT*	*5'-GACCTTTTTTGGCTAAACGCTTTC*
Cxcl13	*5'-CATAGATCGGATTCAAGTTACGCC*	*5'-GTAACCATTTGGCACGAGGATTC*
Cdh1	*5'-GGTCATCAGTGTGCTCACCTCT*	*5'-GCTGTTGTGCTCAAGCCTTCAC*
Cdh2	*5'-CCTCCAGAGTTTACTGCCATGAC*	*5'-CCACCACTGATTCTGTATGCCG*
Cdh3	*5'-CCAGACAAGGAGGACCAGAAGA*	*5'-CAAACTGCTCGTCCTCACGATC*
Cdh5	*5'-GAACGAGGACAGCAACTTCACC*	*5'-GTTAGCGTGCTGGTTCCAGTCA*
Cdh11	*5'-ACACAGGATGGTGTGGTGAAGC*	*5'-CTTGACGGTCACAGTGTCCTTG*
Cdh13	*5'-CGAGAAAGGAGACATTGTCACCG*	*5'-TCAGTCCGACATCCAATCCTGC*
Cdh19	*5'-TGACAGAGAGGTCAGTGCTTGG*	*5'-GAGAGAACTCTGGAGCATTGTCG*
Cdh20	*5'-GGTGCTCTAATGACTGCAAGACC*	*5'-GAGCGTTGTCATTCACATCGAGG*
Cdh24	*5'-CAGAGGTCTTCAGCATCAGCAC*	*5'-GTTGGTGGCTTCTACACGGAAG*
Chil3	*5'-GGTCTGAAAGACAAGAACACTGAG*	*5'-GAGACCATGGCACTGAACG*
Endo1	*5'-GTGCCCATCGTCAACCTGAA*	*5'-GACATCTCTGCCGTCAAAAGAA*
Esm1	*5'-GGCGATAAAACAAGACCAGAAA*	*5'-AAACCAGAGATGAGAAGTGATGG*
Ets2	*5'- GTGGCTTCCAAAAGGAGCAACG*	*5'- TTCACCAGGCTGAACTCGTTGG*
F4/80	*5'-TGTCCTCCTTGCCTGGAC*	*5'-GAGACTTCTGAGCTGACACTGC*
Fnip1	*5'-AGTAATGGGCTGCTTGGAAA*	*5'-CAAAGAAAGAGGCACTCCTGA*
Fli1	*5'- CCATACAGACCAGTCCTCACGA*	*5'- CATGGTCTGTGATCCTCCAAGG*
Hsp90aa1	*5'-GCTTTCAGAGCTGTTGCGGTAC*	*5'-AAAGGCGGAGTTAGCAACCTGG*
Il10	*5'-CGGGAAGACAATAACTGCACCC*	*5'-CGGTTAGCAGTATGTTGTCCAGC*
Il1b	*5'-TGCCACCTTTTGACAGTGATG*	*5'-AAGGTCCACGGGAAAGACAC*
Kdr	*5'- CGAGACCATTGAAGTGACTTGCC*	*5'- TTCCTCACCCTGCGGATAGTCA*
Nos2	*5'-ACATCGACCCGTCCACAGTAT*	*5'-CAGAGGGGTAGGCTTGTCTC*
Nos3	*5'- GCAAGAGGAAGGAGTCTAGCA*	*5'- TCGAGCAAAGGCACAGAAGTGG*
Nrp1	*5'-TCCTGGGAAACTGGTATATCTATGA*	*5'-CATTCCAGAGCAAGGATAATCTG*
Nrp2	*5'-GACGACATTCGGATAAGCACC*	*5'-GTTCCAATCTCCTTCATAGTCATCA*
Pedf	*5'-GCCCTGGTGCTACTCCTCT*	*5'-CGGATCTCAGGCGGTACAG*
Ppargc1a	*5'-GGACATGTGCAGCCAAGACTCT*	*5'-CACTTCAATCCACCCAGAAAGCT*
Ppargc1b	*5'-TCCTGTAAAAGCCCGGAGTAT*	*5'-GCTCTGGTAGGGGCAGTGA*
Plasminogen	*5'-CCTCATAGGCACAACAGGACAC*	*5'-TGGCTGTCAGTGGTATAGCACC*

Pecam1	*5'- CCAAAGCCAGTAGCATCATGGTC*	*5'- GGATGGTGAAGTTGGCTACAGG*
Retnla	*5'-CCAATCCAGCTAACTATCCCTCC*	*5'-ACCCAGTAGCAGTCATCCCA*
Tgfb1	*5'-TGATACGCCTGAGTGGCTGTCT*	*5'-CACAAGAGCAGTGAGCGCTGAA*
Tgfb2	*5'-TTGTTGCCCTCCTACAGACTGG*	*5'-GTAAAGAGGGCGAAGGCAGCAA*
Tgfb3	*5'-AAGCAGCGCTACATAGGTGGCA*	*5'-GGCTGAAAGGTGTGACATGGAC*
Thbs1	*5'-GGGGAGATAACGGTGTGTTTG*	*5'-CGGGGATCAGGTTGGCATT*
Thbs2	*5'-CTGGGCATAGGGCCAAGAG*	*5'-GCTTGACAATCCTGTTGAGATCA*
Tnfa	*5'-TGGAACTGGCAGAAGAG*	*5'-CCATAGAACTGATGAGAGG*
Vash1	*5'-GGCCACCTGGGAAAGGATG*	*5'-CTGTGGGGAGGAAACATCCTT*
Vegfa	*5'-CCCACGACAGAAGGAGAGCAGAAGT*	*5'-CATCAGCGGCACACAGGACGG*
Vegfa120	*5'-CCCACGACAGAAGGAGAGCAGAAGT*	*5'-TTGGCTTGTCACATTTTTCTGGCTT*
Vegfa164	*5'-CCCACGACAGAAGGAGAGCAGAAGT*	*5'-CAAGGCTCACAGTGATTTTCTGGC*
Vegfa188	*5'-CCCACGACAGAAGGAGAGCAGAAGT*	*5'-AACAAGGCTCACAGTGAACGCT*
Vegfb167	*5'-CCTGGAAGAACACAGCCAAT*	*5'-CACCTACAGGTGTCTGGGTTGAGCT*
Vegfb186	*5'-CCAGACAGGGTTGCCATA*	*5'-GCTGGAGTGGGATGGATG*

Uncropped scans for Supplemental Figure. 3

Supplemental Figure 3c

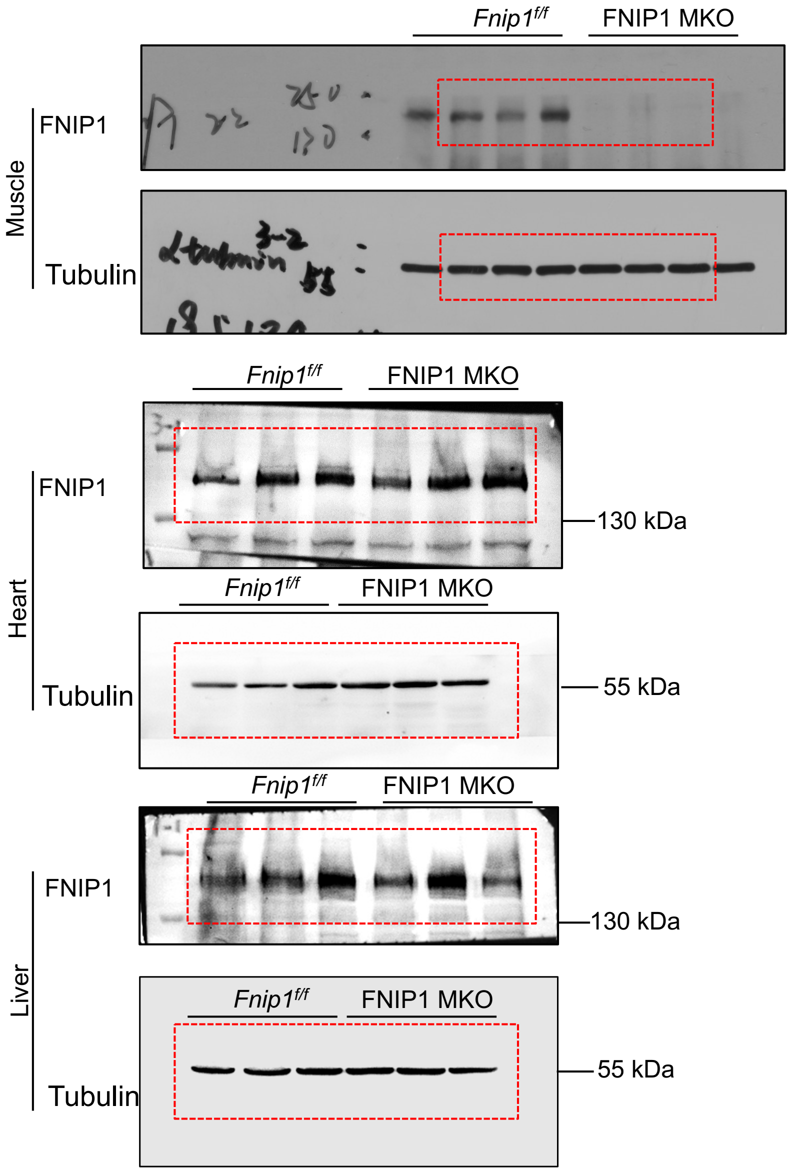

Supplemental Figure 3f

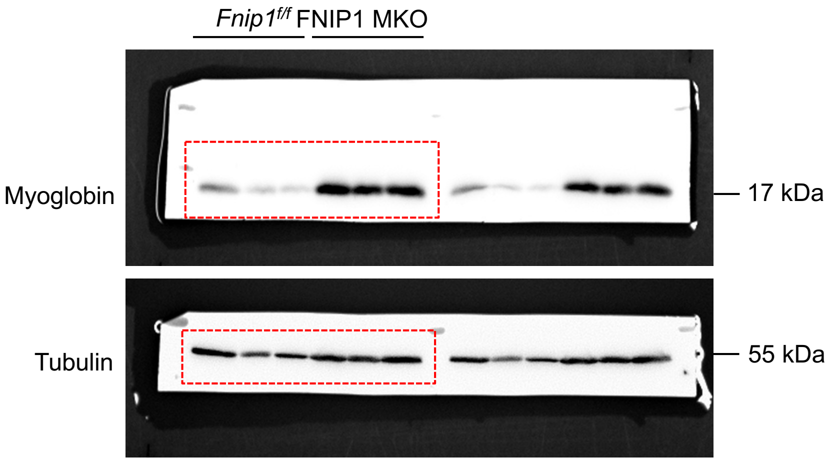

Uncropped scans for Supplemental Figure. 7

Supplemental Figure 7a

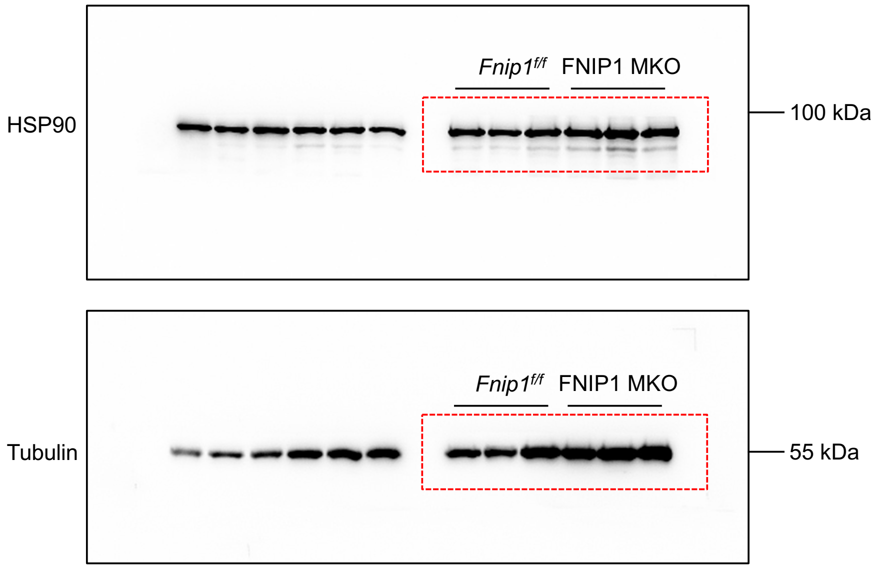

Supplemental Figure 7f

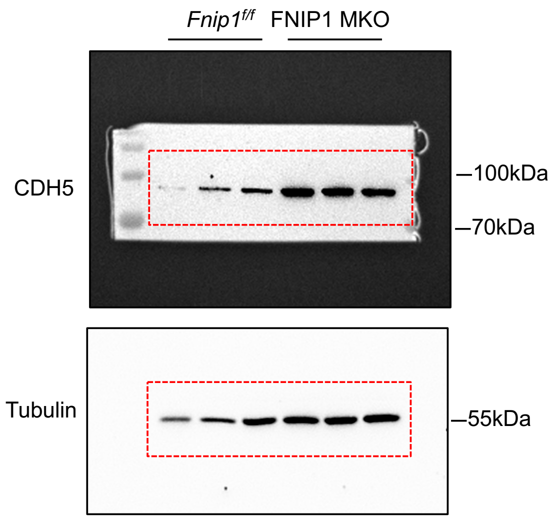

Supplemental Figure 7g

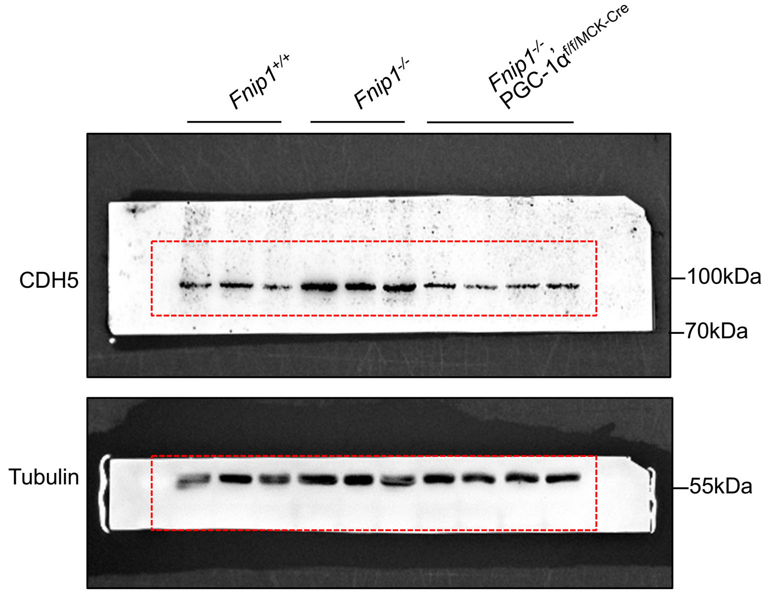

Supplement: Supplementary file 1 — Supplementary Information [file 41467_2023_42690_MOESM1_ESM.pdf]
