# Supplementary material for: FNIP1 abrogation promotes functional revascularization of ischemic skeletal muscle by driving macrophage recruitment
Source: Nat Commun. 2023 Nov 6;14:7136. doi: 10.1038/s41467-023-42690-9 (PMC10628247; doi:10.1038/s41467-023-42690-9)
Supplement: Supplementary file 3 — Reporting Summary [file 41467_2023_42690_MOESM3_ESM.pdf]

nature portfolio

Corresponding author(s): Zhenji Gan

Last updated by author(s): Oct 16, 2023

Reporting Summary

Nature Portfolio wishes to improve the reproducibility of the work that we publish. This form provides structure for consistency and transparency in reporting. For further information on Nature Portfolio policies, see our Editorial Policies and the Editorial Policy Checklist.

Statistics

For all statistical analyses, confirm that the following items are present in the figure legend, table legend, main text, or Methods section.

n/a	Confirmed	
☐	☒	The exact sample size (n) for each experimental group/condition, given as a discrete number and unit of measurement
☐	☒	A statement on whether measurements were taken from distinct samples or whether the same sample was measured repeatedly
☐	☒	The statistical test(s) used AND whether they are one- or two-sided<br>*Only common tests should be described solely by name; describe more complex techniques in the Methods section.*
☒	☐	A description of all covariates tested
☐	☒	A description of any assumptions or corrections, such as tests of normality and adjustment for multiple comparisons
☐	☒	A full description of the statistical parameters including central tendency (e.g. means) or other basic estimates (e.g. regression coefficient) AND variation (e.g. standard deviation) or associated estimates of uncertainty (e.g. confidence intervals)
☐	☒	For null hypothesis testing, the test statistic (e.g. F, t, r) with confidence intervals, effect sizes, degrees of freedom and P value noted<br>*Give P values as exact values whenever suitable.*
☒	☐	For Bayesian analysis, information on the choice of priors and Markov chain Monte Carlo settings
☒	☐	For hierarchical and complex designs, identification of the appropriate level for tests and full reporting of outcomes
☐	☒	Estimates of effect sizes (e.g. Cohen's d, Pearson's r), indicating how they were calculated

Our web collection on statistics for biologists contains articles on many of the points above.

Software and code

Policy information about availability of computer code

Data collection	mRNA-Seq were performed using Illumina HiSeq 4000, and paired-end,150nt reads were obtained from the same sequencing lane; Microscopy pictures were acquired by ZEISS LSM880 (ZEISS); Flow cytometry data were detected by FACS Aria III (BD Bioscience) sorter; The recovery of blood flow was tracked non-invasively by infrared Doppler scanning (Laser Doppler Perfusion Imager System, moorLDI-Mark 2, Wilmington, DE).
Data analysis	The sequencing reads were then aligned to the UCSC mm10 genome assembly using TopHat 2.0.14 with the default parameters. Fragments Per Kb of exon per Million mapped reads (FPKM) were calculated using Cufflinks 2.2.1. The criteria for a regulated gene were a fold change greater than 1.5 (either direction) and a significant P-value (< 0.05) versus control. For pathway analysis, the filtered data sets were uploaded into DAVID Bioinformatics Resources 6.8 to review the bio pathways using the Functional Categories database. The GO analysis “Biological Process_ Direct” term was used to interpret data, and the regulated terms were ranked by P-value. The heat-map analysis of regulated genes was generated by using R software (Version 3.3.2) and ggplot2/gplots package. Image analysis was performed with Image J (version 1.51, https://imagej. nih.gov/). FlowJo_v10.8.1 (Tree Star) was used to visualize flow cytometry data.

For manuscripts utilizing custom algorithms or software that are central to the research but not yet described in published literature, software must be made available to editors and reviewers. We strongly encourage code deposition in a community repository (e.g. GitHub). See the Nature Portfolio guidelines for submitting code & software for further information.

Data

Policy information about availability of data

All manuscripts must include a data availability statement. This statement should provide the following information, where applicable:

- Accession codes, unique identifiers, or web links for publicly available datasets
- A description of any restrictions on data availability
- For clinical datasets or third party data, please ensure that the statement adheres to our policy

The RNA-seq data reported in this paper have been deposited in the Genome Sequence Archive in National Genomics Data Center, China National Center for Bioinformation/Beijing Institute of Genomics, Chinese Academy of Sciences GSA: CRA008213 (https://ngdc.cncb.ac.cn/search/?dbId=gsa&q=CRA008213) and CRA008211 (https://ngdc.cncb.ac.cn/search/?dbId=gsa&q=CRA008211) that are publicly accessible at (https://ngdc.cncb.ac.cn/gsa). Mouse genome (UCSC mm10) (http://www.genome.ucsc.edu). All the source data of this study are provided as Source Data file. Source data are provided with this paper.

Research involving human participants, their data, or biological material

Policy information about studies with human participants or human data. See also policy information about sex, gender (identity/presentation), and sexual orientation and race, ethnicity and racism.

Reporting on sex and gender	N/A
Reporting on race, ethnicity, or other socially relevant groupings	N/A
Population characteristics	N/A
Recruitment	N/A
Ethics oversight	N/A

Note that full information on the approval of the study protocol must also be provided in the manuscript.

Field-specific reporting

Please select the one below that is the best fit for your research. If you are not sure, read the appropriate sections before making your selection.

☒ Life sciences ☐ Behavioural & social sciences ☐ Ecological, evolutionary & environmental sciences

For a reference copy of the document with all sections, see nature.com/documents/nr-reporting-summary-flat.pdf

Life sciences study design

All studies must disclose on these points even when the disclosure is negative.

Sample size	Sample sizes were indicated in the legend of each Figure and Supplementary Figure. No statistical tests were performed to pre-determine sample size. Sample sizes for the skeletal muscle studies were selected due to more than 10 years previous experience demonstrating the minimum number of animals necessary to achieve statistically significant and reproducible results (Gan Z et al. 2011. Genes Dev. PMID: 22135324; Gan Z et al. 2013. J Clin Invest. PMID: 23676496; Liu J et al. 2016. EMBO Mol Med. PMID:27506754; Fu T et al. 2018. Cell Rep. PMID: 29719250; Liu L et al. 2020. J Clin Invest. PMID: 32544095; Xiao L et al. 2021. Plos Genet. PMID: 33780446; He S et al. 2021. J Clin Invest. PMID: 34283807). For experiments involving cell cultures, no sample-size calculation was performed and at least 3 biological replicates per condition were used according to previous studies to enable statistical analysis (Gan Z et al. 2013. J Clin Invest. PMID: 23676496; Xu Z et al. Nat Commun. 2022. PMID: 35173176; Fu T et al. Nat Cell Biol. 2023. PMID: 37217599).
Data exclusions	No data were excluded from the analysis.
Replication	The experimental findings were reliably reproduced, for representative data used for statistical analysis, the number of animals or experiments is described in corresponding figure legends.
Randomization	For all experiments, samples/animals were randomly to experimental groups and processed.
Blinding	In many applications (the fluorescence imaging, histological analysis, confocal microscopy, etc.), acquisition of the data was performed in a blinded fashion. Investigators were not blinded during allocating animal experiment, because the investigators need to conduct genotyping PCRs at the age of 2 weeks for the mice, therefore investigators were not blinded for group identification or genotype identification for the mouse models used in this study.

Reporting for specific materials, systems and methods

We require information from authors about some types of materials, experimental systems and methods used in many studies. Here, indicate whether each material, system or method listed is relevant to your study. If you are not sure if a list item applies to your research, read the appropriate section before selecting a response.

Materials & experimental systems

n/a	Involved in the study
☐	☒ Antibodies
☐	☒ Eukaryotic cell lines
☒	☐ Palaeontology and archaeology
☐	☒ Animals and other organisms
☒	☐ Clinical data
☒	☐ Dual use research of concern
☒	☐ Plants

Methods

n/a	Involved in the study
☒	☐ ChIP-seq
☐	☒ Flow cytometry
☒	☐ MRI-based neuroimaging

Antibodies

Antibodies used

Antibodies directed against PE-anti-CD31 (553373, 1:200 dilution) and CD31 (550274, 1:200 dilution) were from BD Biosciences. Antibodies directed against mPDGFRβ (AF1042, 1:40 dilution) were from R&D Systems, antibodies directed against Brilliant Violet 421-anti-F4/80 (123132, 1:200 dilution), Alexa Fluor 647-anti-CD206 (141712, 1:200 dilution), FITC-anti-CD80 (104705, 1:200 dilution) and APC/Cyanine7-anti-CD45 (103116, 1:200 dilution) were from Biolegend, antibodies directed against Ki67 (ab15580, 1:500 dilution) was from Abcam, antibodies directed against α-tubulin (bs1699, 1:5,000 dilution) were from Bioworld; antibodies directed against NG2 (55027-1-AP, 1:200 dilution), CDH5 (27956-1-AP, 1:1,000 dilution) and Hsp90 (13171-1-AP, 1:1,000 dilution) were from Proteintech; antibodies directed against Myoglobin (sc-25607, 1:1,000 dilution) were from Santa Cruz Biotechnology; antibodies directed against PGC-1α (1:1000 dilution) was developed in the laboratory of Daniel Kelly as described previously (Leone, T.C. et al. PLoS Biol. PMID: 15760270); anti-FNIP1 was developed in the laboratory of Zhenji Gan with the help with Abcam (ab236547, 1:500 dilution). The secondary antibody Alexa Fluor 488 (A-11006, 1:400 dilution), Alexa Fluor 594 (A-21209, 1:400 dilution), Alexa Fluor 568 (A-11011, 1:400 dilution) was from Invitrogen; Alexa Fluor 488 (abs20026, 1:400 dilution) was from Absin.

Validation

Antibodies used in this study are commercial:
1. Rat monoclonal PE anti-mouse CD31 (553373, BD Biosciences, clone number MEC 13.3) manufacturer's website: https://www.bdbiosciences.com/en-us/products/reagents/flow-cytometry-reagents/research-reagents/single-color-antibodies-ruo/pe-rat-anti-mouse-cd31.553373
2. Rat monoclonal anti-mouse CD31 (550274, BD Biosciences, clone number MEC 13.3) manufacturer's website: https://www.bdbiosciences.com/en-us/products/reagents/flow-cytometry-reagents/research-reagents/single-color-antibodies-ruo/purified-rat-anti-mouse-cd31.550274
3. Goat polyclonal anti-mPDGFRb (AF1042, R&D Systems) manufacturer's website: https://www.rndsystems.com/cn/products/mouse-pdgf-rbeta-antibody_af1042.
4. Rat monoclonal Brilliant Violet 421 anti-mouse F4/80 (123132, Biolegend, clone number: BM8) manufacturer's website: https://www.biolegend.com/en-us/products/brilliant-violet-421-anti-mouse-f4-80-antibody-7199?GroupID=BLG5319
5. Rat monoclonal Alexa Fluor 647 anti-mouse CD206 (141712, Biolegend, clone number: C068C2) manufacturer's website: https://www.biolegend.com/en-us/products/alexa-fluor-647-anti-mouse-cd206-mmr-antibody-7427?GroupID=BLG9506
6. Armenian Hamster monoclonal FITC anti-mouse CD80 (104705, Biolegend, clone number: 16-10A1) manufacturer's website: https://www.biolegend.com/en-us/products/fitc-anti-mouse-cd80-antibody-41
7. Rat monoclonal APC/Cyanine7 anti-mouse CD45 (103116, Biolegend, clone number: 30-F11) manufacturer's website: https://www.biolegend.com/en-us/products/apc-cyanine7-anti-mouse-cd45-antibody-2530?GroupID=BLG1932.
8. Rabbit polyclonal anti-Ki67 (ab15580, Abcam) manufacturer's website: https://www.abcam.cn/products/primary-antibodies/ki67-antibody-ab15580.html,
9. Rabbit Polyclonal anti-α-tubulin (bs1699, Bioworld) manufacturer's website: http://www.antibodyreview.com/products/639838.0/Tubulin-alpha-(G436)-polyclonal-antibody-Bioworld-Antibodies-BS1699.html.
10. Rabbit polyclonal anti-NG2 (55027-1-AP, Proteintech) manufacturer's website: https://www.ptglab.co.jp/Products/CSPG4,NG2-Antibody-55027-1-AP.html.
11. Rabbit polyclonal anti-CDH5 (27956-1-AP, Proteintech) manufacturer's website: https://www.ptgcn.com/products/CDH5-Antibody-27956-1-AP.html
12. Rabbit polyclonal anti-Hsp90 (13171-1-AP, Proteintech) manufacturer's website: https://www.ptgcn.com/Products/HSP90-Antibody-13171-1-AP.html
13. Mouse monoclonal Anti-Myoglobin (sc-25607, Santa Cruz Biotechnology, clone number: A-6) manufacturer's website: https://www.scbt.com/zh/p/myoglobin-antibody-a-6
14. Rabbit monoclonal anti-FNIP1 (ab236547, Abcam, clone number EPR20832) manufacturer's website: https://www.abcam.com/products/primary-antibodies/fnip1-antibody-epr20832-bsa-and-azide-free-ab236547.html

Secondary antibodies used in this study:
1. Alexa Fluor 488 Goat anti-Rat IgG (Invitrogen, A-11006, lot: 2416486) manufacturer's website: https://www.thermofisher.cn/cn/zh/antibody/product/Goat-anti-Rat-IgG-H-L-Cross-Adsorbed-Secondary-Antibody-Polyclonal/A-11006;
2. Alexa Fluor 594 Donkey anti-Rat IgG (Invitrogen, A-21209, lot: 2253918) manufacturer's website: https://www.thermofisher.cn/cn/zh/antibody/product/Donkey-anti-Rat-IgG-H-L-Highly-Cross-Adsorbed-Secondary-Antibody-Polyclonal/A-21209;
3. Alexa Fluor 568 Goat anti-Rabbit IgG (Invitrogen, A-11011, lot: 2500544) manufacturer's website: https://www.thermofisher.cn/

cn/zh/antibody/product/Goat-anti-Rabbit-IgG-H-L-Cross-Adsorbed-Secondary-Antibody-Polyclonal/A-11011;
4. Alexa Fluor 488 Donkey anti-Goat IgG (Absin, abs20026, lot: 5920D07) manufacturer's website: https://www.absin.cn/donkey-goat-igg-alexafluor-488/abs20026.html.

Eukaryotic cell lines

Policy information about cell lines and Sex and Gender in Research

Cell line source(s)	HEK293T (CRL-3216) and C2C12 Cells (CRL-1772) were obtained from the American Type Culture Collection, and were cultured at 37°C and 5% CO2 in Dulbecco's modified Eagle's medium supplemented with 10% fetal calf serum, 1,000 U/ml penicillin and 100 mg /ml streptomycin.
Authentication	The cell line was not authenticated.
Mycoplasma contamination	Mycoplasma contamination was not tested in the study.
Commonly misidentified lines (See ICLAC register)	No commercial misidentified cells were used.

Animals and other research organisms

Policy information about studies involving animals; ARRIVE guidelines recommended for reporting animal research, and Sex and Gender in Research

Laboratory animals	All animal performed in this study were approved by the Institutional Animal Care and Use Committee at the Model Animal Research Center (MARC) of Nanjing University (Approval No. GZJ09). The experiments in this manuscript are in compliance with relevant guidelines and ethical regulations. WT C57BL/6J mice purchased from GemPharmatech Co., Ltd (Jiangsu, China) were used for exercise experiments. Fnip1f/f mice were generated by the transgenic mouse facility at the Model Animal Research Center of Nanjing University as previously described26. Briefly, the Cas9 system targeting the exon 6 of the C57BL/6J mice Fnip1 gene was applied. The loxP sites was introduced upstream and downstream of exon 6 (92 bp) of Fnip1 gene by homologous recombination. To generate mice with a myofiber-specific disruption of the Fnip1 allele, Fnip1f/f mice were crossed with mice expressing Cre recombinase under control of a human skeletal actin (HSA) promoter (Jackson Laboratory; stock no. 006139) to achieve myofiber-specific deletion of Fnip1 (FNIP1 MKO). Generation of FNIP1 KO mice have been described elsewhere26. To generate FNIP1 TgKO mice, male Fnip1-/- mice were first bred with female Fnip1MCK-Tg+/- mice to generate Fnip1+/-, Fnip1MCK-Tg+/- males and Fnip1+/- females, which were subsequently intercrossed to produce Fnip1-/-, Fnip1MCK-Tg-/- (FNIP1 KO) and Fnip1-/-, Fnip1MCK-Tg+/- (FNIP1 TgKO) and Fnip1+/+, Fnip1MCK-Tg-/- control littermates. To generate Fnip1-/-, AMPKα1/α2f/f/Myf5-Cre (herein named TKO) mice, male Fnip1-/- mice were first bred with female Ampka1/a2f/f Myf5-cre mice to generate Fnip1+/-, Ampka1/a2f/+ Myf5-cre males and Fnip1+/-, Ampka1/a2f/+ females, which were subsequently intercrossed to obtain male Fnip1+/-, Ampka1/a2f/f Myf5-cre and Fnip1 +/-;Ampka1/a2f/f females. Male Fnip1+/-, Ampka1/a2f/f Myf5-cre mice were finally bred with female Fnip1+/-;Ampka1/a2f/f mice to produce Fnip1-/-, Ampka1/a2f/f and Fnip1-/-, Ampka1/a2f/f Myf5-cre and Fnip1+/+, Ampka1/a2f/f control littermates. To generate Fnip1-/-, PGC-1αf/f/MCK-Cre (herein named DKO) mice, male Fnip1-/- mice were first bred with female Ppargc1af/f MCK-cre mice to generate Fnip1+/-, Ppargc1af/+ MCK-cre males and Fnip1+/-, Ppargc1af/+ females, which were subsequently intercrossed to obtain male Fnip1+/-, Ppargc1af/f MCK-cre and Fnip1+/-, Ppargc1af/f females. Male Fnip1+/-, Ppargc1af/f MCK-cre mice were finally bred with female Fnip1+/-, Ppargc1af/f mice to produce Fnip-/-, Ppargc1af/f and Fnip1-/-, Ppargc1af/f MCK-cre, and Fnip1+/+, Ppargc1af/f control littermates. To collect sera and tissue samples, mice were first anesthetized by isoflurane inhalation and then euthanized by cervical dislocation. Mice between 8 and 14 weeks of age (n = 3 to 11) were used for experiments. Mice were compared to their own littermates and the age of the mice were stated in the Figure legends. Mice were randomly assigned to various analyses. Notably, both male and female mice were used in the study, muscle angiogenesis compared with littermate controls, were similar in female compared with male for FNIP1 KO lines. The animals were maintained with free access to pellet food (XieTong Biology, 1010082), and water in plastic cages at 21 ± 2°C, relative humidity of 55±10% and kept on a 12 h light-dark cycle. All mice are harbored in the specific pathogen-free facility in Nanjing University.
Wild animals	NO
Reporting on sex	Sex was considered in the study, muscle angiogenesis compared with littermate controls, were similar in female compared with male for FNIP1 KO lines.
Field-collected samples	NO
Ethics oversight	The animal use and the experimental protocols were reviewed and approved by the IACUC committees at the Model Animal Research Center (MARC) of Nanjing University.

Note that full information on the approval of the study protocol must also be provided in the manuscript.

Flow Cytometry

Plots

Confirm that:

☒ The axis labels state the marker and fluorochrome used (e.g. CD4-FITC).

☒ The axis scales are clearly visible. Include numbers along axes only for bottom left plot of group (a 'group' is an analysis of identical markers).

☒ All plots are contour plots with outliers or pseudocolor plots.

☒ A numerical value for number of cells or percentage (with statistics) is provided.

Methodology

Sample preparation	Endothelial cells from skeletal muscle were isolated from FNIP1 MKO and FNIP1f/f control littermates, MCK-FNIP1 Tg or NTG mice as described previously10. Briefly, hindlimb muscles were immediately dissected after euthanasia and minced into pieces on ice using a surgical blade. Then, the minced muscles were enzymatically digested in digesting buffer containing 2 mg/mL Collagenase IV (1886986, Gibco) and 2 mg/mL Dispase II (65558200, Roche) and 250 mM CaCl2 in DPBS for 25 min at 37°C with gentle shaking. Next, an equal volume of 20% FBS buffer in DPBS was added to stop the reaction. Thereafter, the suspension was passed through a series of 100-µm cell strainers (352350, Falcon) and 40-µm cell strainers (352340, Falcon) to remove tissue debris. After a series of centrifugation and washing steps, the cell pellets were resuspended in antibody solution with anti-mouse CD31 PE antibody (553373, BD Pharmingen, 1:200) and anti-mouse CD45 APC (103116, Bioligand, 1:200) antibody and incubated for 30 min at dark. After a series of washing steps with FACS wash buffer, the EC were sorted based on CD31+CD45- staining by using FACS Aria III (BD Bioscience) sorter. Data were analyzed using FlowJo 10 software (Tree Star) and the EC cell number was normalized to muscle mass.
Instrument	FACS Aria III (BD Bioscience)
Software	FlowJo 10 software (Tree Star)
Cell population abundance	Putiry of the sorted fractions was confirmed by flow cytometry resulting in a purity of the sorted cells of >95%., as determined by renalysing by FACs a fraction of sorted cells.
Gating strategy	In the figure containing flow cytometry data, cells are initially gated on FSC-A x SSC-A and then gated on singlets by FSC-H vs FSC-A, From singlets, live cells were identified by propidium iodide, endothelial cells were detected by CD31+CD45-.

☐ Tick this box to confirm that a figure exemplifying the gating strategy is provided in the Supplementary Information.
